# *N*-{*N*-[5-(2,4-Dichloro­phen­yl)-1,3,4-thia­diazol-2-yl]carbamo­yl}-2,6-difluoro­benzamide

**DOI:** 10.1107/S1600536809044584

**Published:** 2009-10-31

**Authors:** Peng Wang, Rong Wan, Feng Han, Yao Wang

**Affiliations:** a Department of Applied Chemistry, College of Science, Nanjing University of Technology, No. 5 Xinmofan Road, Nanjing 210009, People’s Republic of China

## Abstract

In the title compound, C_16_H_8_Cl_2_F_2_N_4_O_2_S, the thia­diazole ring makes dihedral angles of 24.94 (14) and 48.11 (14)°, respectively, with the dichloro- and difluoro-substituted benzene rings. An intra­molecular N—H⋯O hydrogen bond results in the formation of a planar (mean deviation 0.0091 Å) six-membered ring. In the crystal structure, mol­ecules form centrosymmetric dimers through pairs of inter­molecular N—H⋯O hydrogen bonds.

## Related literature

For 1,3,4-thia­diazole aryl­urea derivatives, see: Hajjar & Casida (1978 ▶); Leighton *et al.* (1981 ▶); Metcalf *et al.* (1975 ▶). For bond-length data, see: Allen *et al.* (1987 ▶).
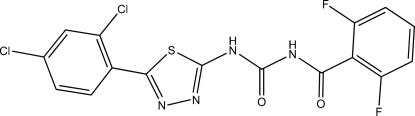


## Experimental

### 

#### Crystal data

C_16_H_8_Cl_2_F_2_N_4_O_2_S
*M* *_r_* = 429.22
Monoclinic, 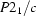

*a* = 8.1600 (16) Å
*b* = 7.6100 (15) Å
*c* = 27.102 (5) Å
β = 92.42 (3)°
*V* = 1681.5 (6) Å^3^
*Z* = 4
Mo *K*α radiation
μ = 0.55 mm^−1^
*T* = 293 K
0.30 × 0.20 × 0.20 mm

#### Data collection

Enraf–Nonius CAD-4 diffractometer
Absorption correction: ψ scan (North *et al.*, 1968 ▶) *T*_min_ = 0.852, *T*_max_ = 0.898
5228 measured reflections
3053 independent reflections
2195 reflections with *I* > 2σ(*I*)
*R*_int_ = 0.034
3 standard reflections every 200 reflections intensity decay: 1%

#### Refinement

*R*[*F* ^2^ > 2σ(*F* ^2^)] = 0.044
*wR*(*F* ^2^) = 0.122
*S* = 1.01
3053 reflections
244 parameters
H-atom parameters constrained
Δρ_max_ = 0.23 e Å^−3^
Δρ_min_ = −0.30 e Å^−3^

### 

Data collection: *CAD-4 EXPRESS* (Enraf-Nonius, 1994 ▶); cell refinement: *CAD-4 EXPRESS*; data reduction: *XCAD4* (Harms & Wocadlo, 1995 ▶); program(s) used to solve structure: *SHELXS97* (Sheldrick, 2008 ▶); program(s) used to refine structure: *SHELXL97* (Sheldrick, 2008 ▶); molecular graphics: *SHELXTL* (Sheldrick, 2008 ▶); software used to prepare material for publication: *SHELXL97*.

## Supplementary Material

Crystal structure: contains datablocks global, I. DOI: 10.1107/S1600536809044584/is2477sup1.cif

Structure factors: contains datablocks I. DOI: 10.1107/S1600536809044584/is2477Isup2.hkl

Additional supplementary materials:  crystallographic information; 3D view; checkCIF report

## Figures and Tables

**Table 1:** Hydrogen-bond geometry (Å, °)

*D*—H⋯*A*	*D*—H	H⋯*A*	*D*⋯*A*	*D*—H⋯*A*
N1—H1*A*⋯O2^i^	0.86	2.07	2.902 (3)	164
N2—H2*A*⋯O1	0.86	1.92	2.607 (3)	136

Symmetry code: (i) 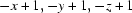.

**Fig. 1.**
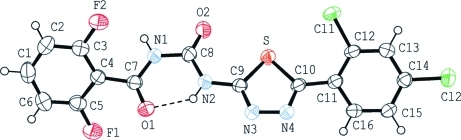
The molecular structure of the title molecule, with the atom-numbering scheme. Displacement ellipsoids are drawn at the 50% probability level. The hydrogen bond is shown as a dashed line.

**Fig. 2.**
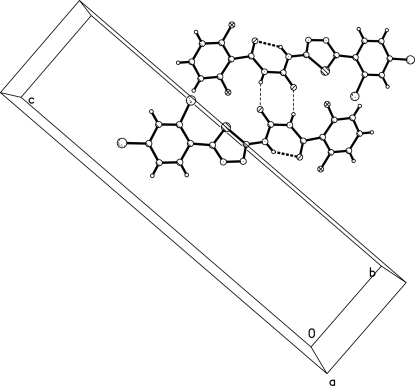
A partial packing diagram of (I). Hydrogen bonds are shown as dashed lines.

## supplementary crystallographic information

### Comment

1,3,4-Thiadiazole aroylurea derivatives are promising and effective insecticides used for the control of insects attacking a wide range of crops. These compounds are generally recognized as insect growth regulators that interfere with chitin synthesis in target pests, causing death or abortive development (Hajjar & Casida, 1978; Leighton *et al.*, 1981). They are considered to be a fourth generation of insecticides with many attractive properties such as high selectivity, low acute toxicity for mammals, and high biological activity, resulting in low application rates (Metcalf *et al.*, 1975).

We report herein the crystal structure of the title compound,(I). In the molecule of the title compound (Fig. 1), the bond lengths (Allen *et al.*, 1987) and angles are within normal ranges. Rings A(C1–C6), B(S/C9/N3/N4/C10) and C(C11–C16) are, of course, planar. The dihedral angle between them is A/B = 47.8 (3)°, A/C=23.1 (3)° and B/C= 24.9 (4)°. The intramolecular N—H···O hydrogen bond (Table 1) results in the formation of one planar six-membered ring D(N2/H2A/O1/C7/N1/C8). They are oriented with respect to the adjacent rings at dihedral angles of A/D= 40.3 (4)°, B/D= 8.6 (4)° and C/D= 17.3 (1)°. So rings B and D are nearly coplanar. In the crystal structure, intermolecular N—H···O hydrogen bonds (Table 1) link the molecules to form a dimeric unit (Fig. 2), in which they may be effective in the stabilization of the structure.

### Experimental

2,6-Difluorobenzoyl isocyanate (14 mmol) was added dropwise to the solution of 5-(2,4-dichlorophenyl)-1,3,4-thiadiazol-2-amine (10 mmol) in toluene under the reflux temperature. The reaction mixture was stirred and refluxed for 5 h. After cooling and filtering, crude compound (I) was obtained. Pure compound (I) was obtained by recrystallization from DMF (15 ml). Crystals of (I) suitable for X-ray diffraction were obtained by slow evaporation of a DMF-H_2_O solution.

### Refinement

H atoms were placed geometrically (C—H = 0.93 and N—H = 0.86 Å) and included in the refinement in riding motion approximation, with *U*_iso_(H) = 1.2*U*_eq_ of the carrier atom.

### Crystal data

**Table:**

C_16_H_8_Cl_2_F_2_N_4_O_2_S	*F*(000) = 864
*M**_r_* = 429.22	*D*_x_ = 1.696 Mg m^−^^3^
Monoclinic, *P*2_1_/*c*	Melting point: 498 K
Hall symbol: -P 2ybc	Mo *K*α radiation, λ = 0.71073 Å
*a* = 8.1600 (16) Å	Cell parameters from 25 reflections
*b* = 7.6100 (15) Å	θ = 10–13°
*c* = 27.102 (5) Å	µ = 0.55 mm^−^^1^
β = 92.42 (3)°	*T* = 293 K
*V* = 1681.5 (6) Å^3^	Block, yellow
*Z* = 4	0.30 × 0.20 × 0.20 mm

### Data collection

**Table:**

Enraf–Nonius CAD-4 diffractometer	2195 reflections with *I* > 2σ(*I*)
Radiation source: fine-focus sealed tube	*R*_int_ = 0.034
graphite	θ_max_ = 25.3°, θ_min_ = 1.5°
ω/2θ scans	*h* = 0→9
Absorption correction: ψ scan (North *et al.*, 1968)	*k* = −4→9
*T*_min_ = 0.852, *T*_max_ = 0.898	*l* = −32→32
5228 measured reflections	3 standard reflections every 200 reflections
3053 independent reflections	intensity decay: 1%

### Refinement

**Table:**

Refinement on *F*^2^	Primary atom site location: structure-invariant direct methods
Least-squares matrix: full	Secondary atom site location: difference Fourier map
*R*[*F*^2^ > 2σ(*F*^2^)] = 0.044	Hydrogen site location: inferred from neighbouring sites
*wR*(*F*^2^) = 0.122	H-atom parameters constrained
*S* = 1.01	*w* = 1/[σ^2^(*F*_o_^2^) + (0.07*P*)^2^] where *P* = (*F*_o_^2^ + 2*F*_c_^2^)/3
3053 reflections	(Δ/σ)_max_ < 0.001
244 parameters	Δρ_max_ = 0.23 e Å^−^^3^
0 restraints	Δρ_min_ = −0.30 e Å^−^^3^

### Special details

**Table:**

Geometry. All e.s.d.'s (except the e.s.d. in the dihedral angle between two l.s. planes) are estimated using the full covariance matrix. The cell e.s.d.'s are taken into account individually in the estimation of e.s.d.'s in distances, angles and torsion angles; correlations between e.s.d.'s in cell parameters are only used when they are defined by crystal symmetry. An approximate (isotropic) treatment of cell e.s.d.'s is used for estimating e.s.d.'s involving l.s. planes.
Refinement. Refinement of *F*^2^ against ALL reflections. The weighted *R*-factor *wR* and goodness of fit *S* are based on *F*^2^, conventional *R*-factors *R* are based on *F*, with *F* set to zero for negative *F*^2^. The threshold expression of *F*^2^ > σ(*F*^2^) is used only for calculating *R*-factors(gt) *etc*. and is not relevant to the choice of reflections for refinement. *R*-factors based on *F*^2^ are statistically about twice as large as those based on *F*, and *R*- factors based on ALL data will be even larger.

### Fractional atomic coordinates and isotropic or equivalent isotropic displacement parameters (Å^2^)

**Table:**

	*x*	*y*	*z*	*U*_iso_*/*U*_eq_	
S	0.24677 (10)	0.99323 (10)	0.44933 (3)	0.0422 (2)	
Cl1	0.07127 (15)	1.02058 (11)	0.34523 (3)	0.0704 (3)	
Cl2	−0.13977 (11)	1.65344 (11)	0.29072 (3)	0.0557 (3)	
F1	0.6408 (2)	0.7102 (3)	0.70051 (7)	0.0601 (5)	
F2	0.4085 (3)	0.2644 (2)	0.60078 (7)	0.0616 (5)	
O1	0.3906 (3)	0.7670 (3)	0.63370 (8)	0.0534 (6)	
O2	0.3952 (3)	0.6786 (3)	0.48524 (7)	0.0584 (7)	
N1	0.4499 (3)	0.6085 (3)	0.56566 (8)	0.0433 (6)	
H1A	0.4957	0.5131	0.5562	0.052*	
N2	0.3126 (3)	0.8663 (3)	0.54369 (9)	0.0419 (6)	
H2A	0.3102	0.8843	0.5750	0.050*	
N3	0.1602 (3)	1.1185 (3)	0.53201 (9)	0.0429 (6)	
N4	0.0980 (3)	1.2319 (3)	0.49659 (9)	0.0433 (6)	
C1	0.6387 (4)	0.2440 (5)	0.71587 (13)	0.0592 (10)	
H1B	0.6765	0.1602	0.7386	0.071*	
C2	0.5527 (4)	0.1908 (4)	0.67364 (13)	0.0526 (9)	
H2B	0.5324	0.0724	0.6675	0.063*	
C3	0.4981 (4)	0.3162 (4)	0.64105 (11)	0.0432 (7)	
C4	0.5219 (3)	0.4949 (4)	0.64808 (10)	0.0372 (7)	
C5	0.6099 (4)	0.5392 (4)	0.69135 (11)	0.0447 (8)	
C6	0.6695 (4)	0.4191 (5)	0.72498 (12)	0.0557 (9)	
H6A	0.7292	0.4546	0.7532	0.067*	
C7	0.4482 (4)	0.6364 (4)	0.61606 (10)	0.0390 (7)	
C8	0.3853 (4)	0.7180 (4)	0.52848 (11)	0.0417 (7)	
C9	0.2414 (3)	0.9916 (4)	0.51268 (10)	0.0363 (7)	
C10	0.1319 (3)	1.1852 (4)	0.45196 (10)	0.0359 (7)	
C11	0.0728 (3)	1.2949 (4)	0.40993 (10)	0.0366 (7)	
C12	0.0397 (4)	1.2367 (4)	0.36183 (11)	0.0381 (7)	
C13	−0.0232 (4)	1.3465 (4)	0.32543 (11)	0.0425 (7)	
H13A	−0.0431	1.3052	0.2934	0.051*	
C14	−0.0561 (4)	1.5183 (4)	0.33714 (10)	0.0395 (7)	
C15	−0.0260 (4)	1.5824 (4)	0.38409 (11)	0.0481 (8)	
H15A	−0.0488	1.6988	0.3916	0.058*	
C16	0.0386 (4)	1.4703 (4)	0.41965 (11)	0.0455 (8)	
H16A	0.0601	1.5134	0.4514	0.055*	

### Atomic displacement parameters (Å^2^)

**Table:**

	*U*^11^	*U*^22^	*U*^33^	*U*^12^	*U*^13^	*U*^23^
S	0.0482 (5)	0.0403 (5)	0.0384 (4)	0.0116 (4)	0.0050 (3)	−0.0003 (3)
Cl1	0.1326 (9)	0.0327 (5)	0.0451 (5)	0.0130 (5)	−0.0047 (5)	−0.0082 (4)
Cl2	0.0709 (6)	0.0489 (5)	0.0469 (5)	0.0135 (4)	−0.0026 (4)	0.0082 (4)
F1	0.0674 (13)	0.0525 (12)	0.0589 (12)	−0.0073 (10)	−0.0146 (10)	−0.0105 (9)
F2	0.0766 (14)	0.0489 (12)	0.0578 (12)	−0.0075 (10)	−0.0145 (10)	−0.0106 (9)
O1	0.0714 (16)	0.0449 (14)	0.0436 (12)	0.0189 (12)	0.0004 (11)	−0.0060 (10)
O2	0.0841 (17)	0.0548 (15)	0.0363 (12)	0.0297 (13)	0.0035 (11)	−0.0017 (10)
N1	0.0530 (16)	0.0385 (15)	0.0382 (13)	0.0141 (12)	−0.0017 (11)	−0.0005 (11)
N2	0.0498 (15)	0.0402 (15)	0.0354 (13)	0.0098 (12)	−0.0024 (11)	−0.0021 (11)
N3	0.0494 (15)	0.0373 (15)	0.0415 (14)	0.0091 (12)	−0.0035 (12)	−0.0026 (12)
N4	0.0510 (16)	0.0396 (15)	0.0390 (14)	0.0107 (12)	−0.0015 (12)	−0.0031 (11)
C1	0.062 (2)	0.064 (3)	0.052 (2)	0.0136 (19)	−0.0012 (18)	0.0170 (18)
C2	0.055 (2)	0.0393 (19)	0.064 (2)	0.0022 (16)	0.0085 (17)	0.0071 (16)
C3	0.0423 (17)	0.0447 (19)	0.0427 (17)	−0.0009 (15)	0.0026 (13)	−0.0031 (14)
C4	0.0361 (15)	0.0392 (17)	0.0363 (15)	−0.0001 (13)	0.0028 (12)	−0.0007 (13)
C5	0.0415 (18)	0.048 (2)	0.0449 (17)	−0.0003 (15)	0.0009 (14)	−0.0045 (15)
C6	0.055 (2)	0.065 (2)	0.0464 (19)	0.0020 (18)	−0.0079 (16)	0.0048 (17)
C7	0.0402 (16)	0.0381 (17)	0.0386 (16)	0.0004 (14)	−0.0009 (13)	−0.0042 (13)
C8	0.0433 (17)	0.0418 (18)	0.0399 (17)	0.0086 (14)	0.0011 (14)	0.0004 (14)
C9	0.0378 (16)	0.0343 (16)	0.0365 (15)	0.0011 (13)	−0.0032 (12)	−0.0050 (13)
C10	0.0385 (16)	0.0279 (15)	0.0410 (16)	0.0011 (13)	−0.0003 (13)	−0.0042 (12)
C11	0.0399 (16)	0.0315 (16)	0.0387 (15)	0.0019 (13)	0.0052 (13)	−0.0024 (13)
C12	0.0456 (17)	0.0281 (16)	0.0408 (16)	0.0000 (13)	0.0056 (13)	−0.0062 (12)
C13	0.0507 (18)	0.0406 (18)	0.0360 (15)	0.0008 (15)	0.0001 (13)	−0.0035 (14)
C14	0.0420 (17)	0.0375 (18)	0.0393 (16)	0.0009 (14)	0.0055 (13)	0.0025 (13)
C15	0.069 (2)	0.0321 (17)	0.0429 (17)	0.0119 (16)	0.0016 (15)	−0.0034 (14)
C16	0.063 (2)	0.0365 (18)	0.0368 (16)	0.0031 (16)	0.0006 (14)	−0.0060 (13)

### Geometric parameters (Å, °)

**Table:**

S—C9	1.719 (3)	C1—H1B	0.9300
S—C10	1.738 (3)	C2—C3	1.362 (4)
Cl1—C12	1.727 (3)	C2—H2B	0.9300
Cl2—C14	1.742 (3)	C3—C4	1.386 (4)
F1—C5	1.347 (4)	C4—C5	1.390 (4)
F2—C3	1.347 (4)	C4—C7	1.494 (4)
O1—C7	1.206 (3)	C5—C6	1.365 (4)
O2—C8	1.216 (3)	C6—H6A	0.9300
N1—C7	1.383 (4)	C10—C11	1.477 (4)
N1—C8	1.394 (4)	C11—C16	1.391 (4)
N1—H1A	0.8600	C11—C12	1.393 (4)
N2—C8	1.348 (4)	C12—C13	1.375 (4)
N2—C9	1.383 (4)	C13—C14	1.375 (4)
N2—H2A	0.8600	C13—H13A	0.9300
N3—C9	1.294 (3)	C14—C15	1.375 (4)
N3—N4	1.372 (3)	C15—C16	1.375 (4)
N4—C10	1.302 (4)	C15—H15A	0.9300
C1—C6	1.376 (5)	C16—H16A	0.9300
C1—C2	1.378 (5)		
			
C9—S—C10	85.91 (14)	N1—C7—C4	116.3 (3)
C7—N1—C8	127.1 (3)	O2—C8—N2	123.3 (3)
C7—N1—H1A	116.5	O2—C8—N1	120.7 (3)
C8—N1—H1A	116.5	N2—C8—N1	115.9 (3)
C8—N2—C9	124.8 (2)	N3—C9—N2	118.5 (2)
C8—N2—H2A	117.6	N3—C9—S	115.7 (2)
C9—N2—H2A	117.6	N2—C9—S	125.7 (2)
C9—N3—N4	111.5 (2)	N4—C10—C11	119.1 (3)
C10—N4—N3	113.0 (2)	N4—C10—S	113.8 (2)
C6—C1—C2	121.1 (3)	C11—C10—S	127.1 (2)
C6—C1—H1B	119.5	C16—C11—C12	116.7 (3)
C2—C1—H1B	119.5	C16—C11—C10	117.3 (3)
C3—C2—C1	118.3 (3)	C12—C11—C10	125.9 (3)
C3—C2—H2B	120.9	C13—C12—C11	122.0 (3)
C1—C2—H2B	120.9	C13—C12—Cl1	116.5 (2)
F2—C3—C2	118.1 (3)	C11—C12—Cl1	121.4 (2)
F2—C3—C4	117.8 (3)	C12—C13—C14	118.9 (3)
C2—C3—C4	123.9 (3)	C12—C13—H13A	120.6
C3—C4—C5	114.7 (3)	C14—C13—H13A	120.6
C3—C4—C7	125.2 (3)	C15—C14—C13	121.5 (3)
C5—C4—C7	119.8 (3)	C15—C14—Cl2	120.5 (2)
F1—C5—C6	117.7 (3)	C13—C14—Cl2	118.0 (2)
F1—C5—C4	118.4 (3)	C14—C15—C16	118.5 (3)
C6—C5—C4	123.9 (3)	C14—C15—H15A	120.8
C5—C6—C1	118.1 (3)	C16—C15—H15A	120.8
C5—C6—H6A	121.0	C15—C16—C11	122.4 (3)
C1—C6—H6A	121.0	C15—C16—H16A	118.8
O1—C7—N1	122.6 (3)	C11—C16—H16A	118.8
O1—C7—C4	121.2 (3)		
			
C9—N3—N4—C10	1.1 (4)	N4—N3—C9—S	−1.6 (3)
C6—C1—C2—C3	−0.2 (5)	C8—N2—C9—N3	172.6 (3)
C1—C2—C3—F2	−177.4 (3)	C8—N2—C9—S	−5.9 (4)
C1—C2—C3—C4	−0.9 (5)	C10—S—C9—N3	1.2 (2)
F2—C3—C4—C5	177.6 (3)	C10—S—C9—N2	179.8 (3)
C2—C3—C4—C5	1.0 (5)	N3—N4—C10—C11	180.0 (3)
F2—C3—C4—C7	3.7 (5)	N3—N4—C10—S	−0.2 (3)
C2—C3—C4—C7	−172.8 (3)	C9—S—C10—N4	−0.5 (2)
C3—C4—C5—F1	178.9 (3)	C9—S—C10—C11	179.3 (3)
C7—C4—C5—F1	−6.9 (4)	N4—C10—C11—C16	23.2 (4)
C3—C4—C5—C6	−0.1 (5)	S—C10—C11—C16	−156.5 (2)
C7—C4—C5—C6	174.2 (3)	N4—C10—C11—C12	−153.1 (3)
F1—C5—C6—C1	−179.9 (3)	S—C10—C11—C12	27.1 (4)
C4—C5—C6—C1	−0.9 (5)	C16—C11—C12—C13	0.2 (4)
C2—C1—C6—C5	1.1 (5)	C10—C11—C12—C13	176.6 (3)
C8—N1—C7—O1	−1.6 (5)	C16—C11—C12—Cl1	−178.2 (2)
C8—N1—C7—C4	178.2 (3)	C10—C11—C12—Cl1	−1.9 (4)
C3—C4—C7—O1	136.8 (3)	C11—C12—C13—C14	−0.8 (5)
C5—C4—C7—O1	−36.8 (4)	Cl1—C12—C13—C14	177.7 (2)
C3—C4—C7—N1	−43.1 (4)	C12—C13—C14—C15	0.6 (5)
C5—C4—C7—N1	143.3 (3)	C12—C13—C14—Cl2	−178.7 (2)
C9—N2—C8—O2	−0.3 (5)	C13—C14—C15—C16	0.0 (5)
C9—N2—C8—N1	179.9 (3)	Cl2—C14—C15—C16	179.3 (2)
C7—N1—C8—O2	179.7 (3)	C14—C15—C16—C11	−0.6 (5)
C7—N1—C8—N2	−0.5 (5)	C12—C11—C16—C15	0.5 (5)
N4—N3—C9—N2	179.8 (3)	C10—C11—C16—C15	−176.2 (3)

### Hydrogen-bond geometry (Å, °)

**Table:**

*D*—H···*A*	*D*—H	H···*A*	*D*···*A*	*D*—H···*A*
N1—H1A···O2^i^	0.86	2.07	2.902 (3)	164
N2—H2A···O1	0.86	1.92	2.607 (3)	136

Symmetry codes: (i) −*x*+1, −*y*+1, −*z*+1.
